# The Impact of Expert Witness Knowledge on Perceived Credibility: Implications for Not Guilty by Reason of Insanity (NGRI) Endorsement

**DOI:** 10.3390/bs15101411

**Published:** 2025-10-16

**Authors:** Cayla F. Cain, Olivia K. H. Smith

**Affiliations:** 1 Department of Psychology, University of South Carolina Aiken, Aiken, SC 29801, USA; 2 Department of Criminal Justice & Sociology, University of Wyoming, Laramie, WY 82071, USA; osmith3@uwyo.edu

**Keywords:** expert witness, credibility, insanity plea, knowledge, individual attitudes

## Abstract

Previous research demonstrates that expert witness education and experience have an influence on mock juror perceptions of credibility. However, whether this relationship extends to cases involving the insanity defense remains unclear, leaving an important gap in the literature given the high stakes of such trials. The current study used an experimental design to examine the impact of expert witness knowledge (high vs. low) on perceived credibility and subsequent NGRI endorsement. Participants (*N* = 425) read a case summary and the credentials and testimony of the expert witness, completed questionnaires, and reported the likelihood that they would endorse NGRI for the defendant. Results indicated that, regardless of expert witness testimony, prior attitudes about the insanity defense (IDA-R) predicted NGRI endorsement. Specifically, positive attitudes towards the insanity defense resulted in an increased likelihood of NGRI endorsement. These findings underscore that juror attitudes toward the insanity defense, rather than expert witness characteristics, may be the decisive factor shaping NGRI endorsement. This highlights the need for courts to consider such attitudes during jury selection in NGRI cases, paralleling the practice of death qualification in capital trials.

## 1. Introduction

On 20 June 2001, Andrea Yates drowned her five children in the family bathtub and was charged with capital murder. She pled not guilty by reason of insanity (NGRI) due to her postpartum psychosis (McLellan, 2006). For a defendant to establish a defense of insanity, it must be proved that at the time of the offense, they were unable to think or reason rationally and did not understand that their actions were wrong (FindLaw, 2019). In Yates’ case, the jury ultimately accepted her plea (State of Texas v. Andrea Yates, 2005). While this case was popular in the media, Yates was not the first to pursue such a defense. Historically, other defendants have pursued NGRI, such as in the following cases: State of Arizona v. Steinberg (1982), Commonwealth of Virginia v. Bobbit (1994), and United States v. Hinckley (1981). Although these cases have resulted in both successful and unsuccessful attempts at NGRI, they all have one commonality—the presence of an expert witness at trial.

Expert witnesses are appointed by the judge, prosecution, or defense to reliably educate jurors on their field of expertise. This ensures the jury can make an informed decision regarding the defendant (Hudachek & Quigley-McBride, 2022). However, expert witnesses often vary in their knowledge level, including expertise, education, credentials, training, and experience, and previous research demonstrates that jurors’ perceptions of these domains influence expert witness’ perceived credibility (Cramer et al., 2014; LaBat et al., 2023; Neal et al., 2012b; Overton, 2018; Parrott et al., 2015a, 2015b; Titcomb, 2010; Younan & Martire, 2021).

Credibility, when applied to an expert witness, has been conceptualized according to four domains: knowledge, trustworthiness, likeability, and confidence (Brodsky et al., 2010). Given the often-complicated nature of testimony and legal proceedings (i.e., the ambiguity of the NGRI verdict), jurors often bypass the content of expert testimony and rely on external sources as a low-effort way of understanding complex concepts (Flick et al., 2022; Koehler et al., 2016). For example, one external source that scholars highlight is expert witness knowledge as defined by their level of experience and education in the field. Past research shows that expert witness education and experience have an influence on mock juror perceptions of credibility; however, the research conclusions are mixed (Ferreria & Wingrove, 2024; Parrott et al., 2015a, 2015b). The implications of these differences are essential to understand, as jurors make life-altering decisions that determine the fate of defendants at trial (Green & Folingstad, 2009; Hudachek & Quigley-McBride, 2022; Parrott et al., 2015b). Therefore, it is imperative to understand the extent to which expert witness knowledge and their perceived credibility impact individuals’ decision-making. To date, this research has been examined in the context of capital sentencing, for which the verdict options are either the death penalty or life imprisonment (Cramer et al., 2014; Neal et al., 2012b; Parrott et al., 2015a, 2015b; Younan & Martire, 2021). Additionally, expert credibility has been examined in the context of civil trials (Flick et al., 2022), a child sexual abuse case (Ferreria & Wingrove, 2024), and other criminal cases such as theft (LaBat et al., 2023) and assault (Overton, 2018). Thus far, research has not examined perceived expert witness credibility in cases involving a defendant pleading insanity, a context that differs markedly from other criminal trials and places expert witnesses in a crucial role of educating jurors on complex mental disease. Accordingly, the present study investigates how expert witness knowledge shapes public perceptions of credibility and NGRI endorsement.

## 2. Insanity Defense and Mental Illness

Criminal law assumes that individuals are rational, moral agents with free will and thus are responsible for their actions and consequences (Memon, 2006). This issue of sanity became apparent in the seminal M’Naghten case, where a jury found Daniel M’Naghten not guilty by reason of insanity because he was under the influence of delusion at the time of the crime and could not distinguish between right and wrong (Memon, 2006). This court case set the precedent to investigate a defendant’s state of mind if there is proper reason to believe their sanity may have been compromised at the time of the offense.

Less than 1% of criminal defendants use the insanity defense, and only a fourth of those defendants are successful (King, 2024; Runions, 2023). In the United States, that translates to around thirty successful NGRI defenses each year (King, 2024; Runions, 2023). Thus, NGRI is an extremely difficult verdict to prove and requires substantial evidence to support the defense. However, the United States’ approach to NGRI cases is unique when compared to other countries. Specifically, juries determine a defendant’s sanity at the time of the offense through the evidence presented by an expert witness (i.e., psychologist). Each side, the defense and the prosecution, typically appoints its own expert to offer opinions on the defendant’s mental state. In contrast, judges in other countries determine criminal responsibility in cases where a defendant is pleading insanity. Expert witness evaluations are submitted in written form, which the judge reviews and considers when rendering a decision. According to an international review of forensic assessment, countries with formal procedures such as mandated report formats, transparent evaluative criteria, and required expert qualifications produce higher-quality forensic assessments (Pouls et al., 2022). These systems reduce variability in expert opinions and diminish the influence of superficial factors such as presentation style or perceived credibility. This stands in contrast to the U.S. adversarial jury models, where jurors may focus more on how testimony is delivered than on its content. Given this international difference in legal proceedings, the focus of the present study will be on the United States.

Mental state at the time of the offense evaluations, which forensic psychologists often conduct, strive to determine whether NGRI is appropriate for a particular defendant. Forensic psychologists, or expert witnesses, are trained to be unbiased in their evaluations. However, research demonstrates that even with this training, forensic psychologists experience biases during assessment and decision-making (Buongiorno et al., 2025), suggesting biases are difficult to overcome even for those in professional roles. Therefore, lay people may be even more susceptible to biases given their lack of training. For example, individuals hold many different viewpoints that influence how they perceive defendants attempting to plead NGRI, such as biases regarding mental illness and criminal responsibility (Parrott et al., 2015b), the presence of and duration of deliberation (Parrott et al., 2015b), and the severity of the crime committed by the defendant (Adjorlolo et al., 2019; Green & Folingstad, 2009; Hudachek & Quigley-McBride, 2022; LaBat et al., 2023; de Vel-Palumbo et al., 2022; Parrott et al., 2015b; Haroon et al., 2023). These findings suggest that case facts are not the only contributing factor to the outcome of legal cases. Rather, it may be the *perception* that mock jurors develop about an expert witness that impacts verdict decisions. However, these results could also be due to how individuals conceptualize defendants pleading insanity.

For instance, several studies have utilized different measures to examine the effect of views toward mental illness (Beliefs Toward Mental Illness Scale—BMI; Hirai & Clum, 2000) and insanity (Insanity Defense Attitudes Scale—Revised—IDA-R; Skeem et al., 2004), both resulting in similar findings. For example, one study found that when participants were assessed based on their attitudes towards the insanity defense (IDA-R), there was a negative correlation between juror attitudes (i.e., insanity is an excuse for criminal activity) and the likelihood of rendering an NGRI verdict (Green & Folingstad, 2009; Louden & Skeem, 2007; Titcomb, 2010). It has also been demonstrated that the more that an individual views those with mental illness as dangerous (BMI subscale), the more likely they are to be punitive in their verdict decisions (Hudachek & Quigley-McBride, 2022). Given the ambiguity of NGRI verdicts compared to verdicts of guilty or not guilty, research also demonstrates that jurors may use the NGRI verdict as a compromise, with the assumption that the insanity attached to the verdict diminishes criminal responsibility (Finkel, 1995).

Beyond differences in NGRI conceptualizations, attitudinal changes about NGRI also occur among specific subsets of individuals. For instance, individuals who are more likely to be in favor of an NGRI verdict have at least a college education, are liberal, have a favorable view of mental illness (i.e., not stigmatized, anyone’s fault, or in their control), and have previously served as a juror in a criminal case (Butler, 2006). Given the variability across individual perceptions of NGRI and the complexity of establishing mental status at the time of the offense, expert witnesses serve an important role in educating the jury.

## 3. Expert Witnesses

Expert witnesses, such as forensic psychologists, are a key part of determining the validity of a defendant’s insanity defense. However, determining a verdict based on whether the defendant meets the legal definition of insanity is a complicated task for the jury. Research has shown that while expert witness’ credentials and expertise level are relevant in assessing expert witness credibility, they can distract from the evaluation of testimony strength (i.e., scientific merit) (Petty & Cacioppo, 1986; Ratneshwar & Chaiken, 1991; Salerno et al., 2017). One study found that even when the method used by an expert to evaluate evidence was scientifically valid, individuals tended to rely on the experience and background of the expert to determine how much weight to give to that evidence (Koehler et al., 2016). Since the purpose of expert witness testimony is to educate the jury on a specialized subject, a juror’s primary focus should be on the content of the testimony and not the expert’s education and credentials. However, many individuals do not actively put in the effort to understand the content of the testimony, and instead rely on outside factors, which can diminish the importance of testimony content and affect the chosen verdict (Salerno et al., 2017). Moreover, jurors are given little to no opportunity to ask clarifying questions based on the information the expert witness presents, so they are more likely to rely on their own potentially inaccurate interpretations of the evidence. This cognitive process is best explained by The Elaboration Likelihood Model.

## 4. The Elaboration Likelihood Model

The Elaboration Likelihood Model (ELM) is a dual process model that consists of central cues (i.e., message content) and peripheral cues (i.e., message source) as a way in which individuals process information (Petty & Cacioppo, 1986). ELM states that an individual may process both central and peripheral cues simultaneously, though one route typically dominates, depending on motivation and ability to engage with the message (Petty & Cacioppo, 1986). Because central cues require a conscious effort to understand the subject matter, using this type of information requires more cognitive resources. In contrast, peripheral cues require less conscious effort and work best for efficient, quick processing (Petty & Cacioppo, 1986).

There are three circumstances under which the peripheral route of processing information dominates in juror decision-making: if the individual lacks the ability to understand content (Brodsky et al., 2010; Koehler et al., 2016; Petty & Cacioppo, 1986), has no strong attitude towards the issue (Petty & Cacioppo, 1986), has to process jargon-rich language (Petty & Cacioppo, 1986; Salerno et al., 2017), and has no personal stake in the matter (Overton, 2018; Petty & Cacioppo, 1986). Factors that make an individual *less* likely to use peripheral processing include the expert witness’ use of simple language as opposed to jargon (Salerno et al., 2017), if the topic the expert witness is testifying about has high personal relevance to jurors (Greenberg & Wursten, 1988), if jurors have the knowledge and capacity to understand the content (Petty & Cacioppo, 1986), and if the expert witness’s argument is clear, logical, and easy to follow (Petty & Cacioppo, 1986). In summary, there are many factors at play when processing information in a courtroom, including individual factors, central cues, and peripheral cues. Arguably, one of the most researched peripheral cues in courtroom testimony is the credibility of the expert witness.

## 5. Expert Credibility

Previous research has explored the relationship between information processing, both source and content-oriented, and juror perceptions in the context of expert witness credibility. Brodsky et al. (2010) found that a high rating of perceived deceitfulness of the expert witness (i.e., less eye contact, stiffness, nervousness, excessive gesturing, and swallowing) was associated with a lower credibility score. Additionally, if a mock juror perceives the expert witness as pompous, it is associated with lower credibility (Parrott et al., 2015b). Another study found that expert witnesses perceived as being more relaxed, extroverted, and positive were rated by mock jurors as being more credible (Miller & Burgoon, 1982).

Previous literature examining the relationship between juror perceptions of an expert witness and their credibility has been based on factors outlined in the Witness Credibility Model (Brodsky et al., 2010; Parrott et al., 2015a, 2015b). Building on this model, the Witness Credibility Scale (WCS) has been reliably used in this line of research (*α* = 0.95) and consists of the following subscales: trustworthiness (*α* = 0.93), likeability (*α* = 0.86), confidence (*α* = 0.89), and knowledge (*α* = 0.86) (Brodsky et al., 2010). The subscale of expert witness knowledge is “the degree to which an expert is perceived to be well-informed, competent, or perceptive and to possess or exhibit intelligence, insight, understanding, or expertise” (Neal et al., 2012b, p. 490), and it has been of particular interest to researchers due to ease of manipulation. In other words, manipulation of knowledge can occur without a video or audio portrayal of expert testimony, but it is difficult to portray aspects of the WCS that are more dependent on visual depiction, such as likeability and confidence (e.g., facial expressions and body language). Research shows these subscales predict expert credibility in combination and separately. For example, a mock juror’s higher ratings of knowledge (Greenberg & Wursten, 1988; Koehler et al., 2016; LaBat et al., 2023; Neal et al., 2012b; Parrott et al., 2015a, 2015b; Younan & Martire, 2021), likeability (Cramer et al., 2014; LaBat et al., 2023; Neal et al., 2012b; Parrott et al., 2015a, 2015b; Younan & Martire, 2021), confidence (Brodsky et al., 2010; Cramer et al., 2014; LaBat et al., 2023; Parrott et al., 2015a, 2015b; Younan & Martire, 2021), and trustworthiness (Greenberg & Wursten, 1988; Hurwitz et al., 1992; LaBat et al., 2023; Parrott et al., 2015a, 2015b; Younan & Martire, 2021) have all been associated with higher ratings of credibility attributed to an expert witness.

Neal et al. (2012b) examined the relationship between manipulated knowledge (high/low) and likeability (high/low) on expert witness credibility. Results indicated a significant relationship in that the expert with high knowledge was viewed as more credible, while the expert with low knowledge was perceived as less credible. Additionally, it was found that likeability seemed to boost the effect of credibility for both high and low knowledge experts (Neal et al., 2012b; Younan & Martire, 2021). Another study expanded on these findings by examining the main effect of knowledge (high/low; manipulation from Neal et al., 2012b) on mock juror perceptions of expert credibility, agreement with the expert, and sentencing in a capital murder trial (Parrott et al., 2015b). Results indicated that knowledge had a significant positive effect on expert witness credibility and no significant relationship with the sentencing outcome (Parrott et al., 2015b). However, the relationship between knowledge and likeability was complex and may vary depending on the context. Specifically, likeability was shown to moderate the effect of knowledge on credibility, such that the impact of knowledge was enhanced or diminished depending on the expert’s perceived likeability (Parrott et al., 2015b).

Furthermore, Koehler et al. (2016) found that higher mock juror ratings of the more experienced expert witness (i.e., years of experience) were significantly associated with more perceived strength of the evidence (i.e., fingerprint analysis) when compared to the less experienced expert witness. That same study conversely found that the expert experience level did not affect mock jurors’ perception of defendant guilt. Additionally, research has found that actual jurors endorse expert witness knowledge (i.e., training and experience) as one of the most important factors towards credibility ratings, with more emphasis placed on experience (i.e., years in professional career) rather than training (i.e., academic history) (Blackwell & Seymour, 2015; McCarthy Wilcox & Nicdaeid, 2018). Given this distinction between training and experience as separate operational definitions for expert witness knowledge, Ferreria and Wingrove (2024) sought to determine whether expert witness training or experience was more influential in juror perceptions of credibility and subsequent verdict decisions. In their study, the expert witness was a forensic nurse on a child sexual abuse case. Unlike previous research on expert witness knowledge, the researchers manipulated experience and training separately and dichotomously (high/low). Results showed that both expert training and experience influenced credibility ratings, and it was the first study to show that the two factors can separately and *comparably* impact perception (Ferreria & Wingrove, 2024). However, they found that training and experience did not influence mock juror verdict preferences, but that expert witness credibility did. More specifically, the higher the perceived credibility of an expert witness, the more likely it was that mock jurors would render a guilty verdict (Ferreria & Wingrove, 2024). While the relationship between expert credibility and verdict is mixed regarding the previous literature, this study suggests it is important to study credibility in different contexts. While some research demonstrates there is no relationship between credibility and verdict (Neal et al., 2012a, 2012b), context seems to play a role in Ferreria and Wingrove’s (2024) study (i.e., child sexual abuse case). Changing the context, such as an insanity defense case, may impact the relationship among these variables in a novel way. However, there is limited research on the relationship between expert witness’ perceived credibility and NGRI.

## 6. The Current Study

Given the limited research on the relationship between expert witness knowledge, expert witness’ perceived credibility, and NGRI endorsement, the purpose of the current study is to explore these variables together and determine whether a significant association can be identified. Additionally, the evidence in NGRI cases involves complex concepts, such as the defendant’s mental status, which requires expert guidance for laypeople to understand. If individuals are relying on an expert’s credentials (e.g., degree, experience) rather than the content of their testimony, this could undermine the fairness and accuracy of court processes, and legal decisions may then reflect status-based heuristics rather than reasoned evaluation. This can distort justice, especially in cases where the expert with “less prestige” may provide scientifically valid testimony.

The basis of the current study was adapted from Parrott et al. (2015b), which manipulated the level of expert witness knowledge (high/low) and measured mock juror perceptions of credibility, level of agreement with the expert witness, and verdict (guilty/not guilty) in the context of capital sentencing (i.e., life without the possibility of parole or death). The current study will extend this research by examining the effect of expert witness knowledge on public perceptions of expert credibility and the extent of NGRI endorsement in the context of an insanity defense case. Based on previous research, we hypothesize the following:

**Hypothesis 1 (main effect):**  *We predict that participants exposed to the high knowledge expert witness will perceive the expert as more credible than participants exposed to the low knowledge expert witness.*

**Hypothesis 2 (main effect):**  *We predict that participants exposed to the high knowledge expert witness will be more likely to endorse NGRI than participants exposed to the low knowledge expert witness.*

**Hypothesis 3 (main effect):**  *We predict that participants with more positive attitudes towards the insanity defense (IDA-R), will be more likely to endorse NGRI compared to those with more negative attitudes.*

**Hypothesis 4 (interaction effect):**  *We predict that the relationship between expert witness knowledge and NGRI endorsement will be moderated by IDA-R scores. Specifically, we hypothesize that in the high knowledge condition, participants will be more likely to endorse NGRI compared to the low knowledge condition, but only for those with lower IDA-R (i.e., supportive/neutral attitudes) scores.*

Prior to testing the hypotheses, we conducted a pilot study to determine the strength of the manipulations we used in the main study.

## 7. Pilot Study

### 7.1. Method

#### 7.1.1. Participants and Design

Participants (*N* = 150) were recruited through Prolific Academic (https://prolific.com/) [2024], an online recruitment platform used in academic research to obtain high-quality, prescreened samples for online studies, and ranged in age from 18 to 72 years old (*M* = 36.49, *SD* = 12.08). They were 85% White, 59.5% female, 31.3% male, and 1.2% non-binary. The design was experimental and quantitative, and participants were randomly assigned to either the high knowledge condition (*N* = 76) or the low knowledge condition (*N* = 74). Aside from these manipulations, all participants read the same case summary and expert witness testimony. The measured variable of interest was perceived knowledge of the expert witness providing the testimony.

#### 7.1.2. Materials

**Case Summary:** Participants read a case summary describing an alleged homicide that occurred the night of 23 October 2018. The vignette case summary was based on materials from past research (Hudachek & Quigley-McBride, 2022) but adapted to the defendant having a history of mental illness in a homicide case (see Appendix A). The defendant in the case summary, Mr. Ralph Brown, is a 33-year-old male diagnosed with schizophrenia and a history of hospitalizations due to this diagnosis. He claims to have murdered his ex-girlfriend’s boyfriend with a gunshot to the head due to hearing voices commanding him to do so. The expert witness’ credentials are manipulated to reflect high- and low-level knowledge while keeping the testimony identical across conditions.

**Expert Witness Manipulation:** The knowledge manipulation in this study was adapted from Parrott et al. (2015b) (see Appendix B). For the current study, we operationalized high knowledge as a forensic psychologist with a doctorate degree (PhD), who has 10 years of education, is board-certified by the American Board of Forensic Psychology, has over 20 years of experience, has conducted around 4000 forensic assessments and published 50 research publications, and who has testified in many court cases and conducted many interviews with the defendant prior to testifying. The low knowledge expert witness was defined as a forensic psychologist with a master’s degree (M.S.), who has 6 years of education and 2 years of experience in the field, has conducted around 100 forensic assessments, has four research publications, has testified in a handful of cases, and only conducted a few interviews with the defendant before testifying. Both expert witnesses, however, had identical testimonies, in which they explained the nature and implications of Mr. Brown’s diagnosis of schizophrenia. They testified that his delusions caused him to not be of sound mind and incapable of rational thought at the time of the crime, endorsing an NGRI plea (see Appendix C).

#### 7.1.3. Measures

**Survey Questions:** Participants responded to several factual questions regarding the expert witness’ credentials to assess the knowledge manipulation. This included the years of education, years of field experience, number of research publications, and number of forensic assessments conducted. For each question, there was a factually correct answer. They were also asked to identify if the expert witness was board-certified. To assess the effectiveness of the knowledge manipulation, participants responded to a single-item question, “What was your perception of the knowledge of the expert witness in the case you read?” on a 7-point Likert scale of 1 (*not at all knowledgeable*) to 7 (*extremely knowledgeable*). Participant responses ranged from 3 to 7, with an average of 5.99 (*SD* = 1.02).

#### 7.1.4. Procedure

Once participants volunteered for the study on Prolific, they were redirected to a Qualtrics survey containing the study and informed that they would be reading a case summary of an individual accused of murder and answering questions about the testimony and the case. Upon providing online consent to participate, participants read the case summary, their assigned expert witness’ credentials and testimony, and provided demographic information. Participants responded to the questions in the order described above and were compensated $1.25 for approximately 8 minutes of their time.

## 8. Results

### Knowledge Analysis

An independent samples t-test was conducted to compare the mean scores of the high knowledge condition and the low knowledge condition. Results indicated a significant difference between the two groups, *t*(148) = 3.66, *p* < 0.001, *d* = 0.60. Those in the high knowledge condition (*M* = 6.28, *SD* = 0.89) reported significantly higher knowledge than those in the low knowledge condition (*M* = 5.69, *SD* = 1.07). Based on the analysis of the factual and perception questions concerning the experts’ credentials, the knowledge manipulation was successful in both conditions. Specifically, participants were asked the following information about the expert witness: number of years of education they had (high knowledge % correct = 68.4; low knowledge % correct = 89.2), number of years in the field (high knowledge % correct = 53.3; low knowledge % correct = 64.9), number of published research publications (high knowledge % correct = 44.7; low knowledge % correct = 68.9), number of forensic assessments administered (high knowledge % correct = 43.4; low knowledge % correct = 73.0), type of degree they had (high knowledge % correct = 84.2; low knowledge % correct = 83.8), and if they were board-certified (high knowledge % correct = 96.1; low knowledge % correct = 29.7).

## 9. Discussion

The purpose of the pilot study was to ensure that the knowledge manipulation was interpreted by participants as intended. In other words, the study sought to find a significant difference between perceptions of the high and low knowledge expert conditions. Results indicated that the high knowledge expert was perceived as more knowledgeable than the low knowledge expert. Given these results, we use the piloted case materials and manipulations to test our hypotheses.

## 10. Main Study

### 10.1. Method

#### Participants and Design

Participants (*N* = 425) were recruited via Prolific Academic (https://prolific.com/) [2024] to complete the study online on their mobile phone or computer. Inclusion criteria specified that participants must be at least 18 years old and be a United States citizen. The participants ranged in age from 18 to 79 years old (*M* = 43.45; *SD* = 15.75). They were 61.9% White, 12.2% Black, 9.4% Hispanic/Latino, 8.7% Asian, 0.9% Native American, 0.7% Middle Eastern, 4.9% were two or more races, and 1.2% other. The participants were 45.9% male, 52.5% female, 0.9% nonbinary/nonconforming, and 0.7% transgender. The participants’ political affiliations are as follows: 13.9% very liberal, 27.5% liberal, 29.2% moderate, 21.4% conservative, and 8.0% very conservative. The majority of participants had some level of college education, with most having at least a four-year degree (36.0%), and 14.6% having graduated from high school. Using the “pwr” package (Champely, 2020) in R 4.0.2 (R Core Team, 2024), an a priori power analysis was conducted, which indicated that a sample of 428 would be sufficient to detect a small to medium effect size (*d* = 0.20) with sufficient power (0.90). We are following a small to medium effect size as our study aims to compare it to data from past research (Neal et al., 2012b; Parrott et al., 2015b), which identified a small effect size in their results.

A between-subjects, experimental design was used to determine the extent to which expert witness knowledge (high/low) affects perceived expert witness credibility and NGRI endorsement.

### 10.2. Materials

#### 10.2.1. Stimuli

The case summary in this study replicated the version used in the pilot study (see Appendix A).

**Manipulation:** After reading the case summary, participants were randomly assigned to either read the credentials of the high knowledge or low knowledge expert witness. This manipulation was identical to the manipulation from the pilot study.

#### 10.2.2. Measures

**Witness Credibility Scale (WCS):** The WCS (Brodsky et al., 2010) was used to assess expert witness credibility. It is a 20-item scale that is rated using a 10-point Likert scale from 1 (*dishonest*, *uninformed*, etc.) to 10 (*honest*, *informed*, etc.). The minimum score on this assessment is 20, and the maximum score is 200 (Goodman-Delahunty et al., 2021). The higher the score, the more credible the witness. The credibility score is measured across four domains: likeability (*α* = 0.90), trustworthiness (*α* = 0.95), confidence (*α* = 0.92), and knowledge (*α* = 0.93) (Parrott et al., 2015a, 2015b). Brodsky et al. (2010) conducted a Pearson correlation coefficient test on this scale and found support for construct validity with all four subscales. For example, knowledge was highly positively correlated with competence (0.709) and efficiency (0.786) but had high negative correlations with shyness (−0.472) and phoniness (−0.489). Furthermore, the overall credibility scores were strongly positively correlated with competence (0.702) and efficiency (0.752) (Brodsky et al., 2010). Since the facet of interest is knowledge in the current study, subscales are calculated individually and together for a total credibility score (see Appendix D). The WCS demonstrated excellent internal consistency in the current study, with Cronbach’s *α* = 0.97. Total credibility scores for the current sample ranged from 20 to 200 (*M* = 150.69, *SD* = 28.84).

**Insanity Defense Attitude-R (IDA-R):** The IDA-R (Skeem et al., 2004) is a 19-item survey that measures an individual’s perceptions of the NGRI defense on a 5-point Likert scale from 1 (*disagree completely*) to 5 (*agree completely*). The purpose of using this measure is to be informed about the extent to which participants view the insanity defense as legitimate and how that informs their NGRI endorsement. The scale has been validated against previous measures of attitudes toward the insanity defense (Cutler et al., 1992; Hans, 1986; Roberts & Golding, 1991); this validation resulted in two factors, strict liability (*α* = 0.90) and injustice and danger (*α* = 0.80) (Skeem et al., 2004). Strict liability is defined as the extent to which individuals believe in the relation between severe mental illness and impaired decision-making and control. The injustice and danger factor measures the extent to which individuals believe that the NGRI defense is overused (Skeem et al., 2004). Additionally, it was found that the subscale of strict liability was strongly predictive of individual case judgments, while the subscale of injustice and danger was moderately predictive. Higher scores on this scale indicate more negative attitudes toward the insanity defense. The minimum score on this assessment is 19, and the maximum score is 95. The IDA-R demonstrated excellent internal consistency in the current study, Cronbach’s *α* = 0.92. Scores for the current sample ranged from 23 to 91 (*M* = 54.00, *SD* = 13.40), demonstrating that our sample held moderately negative attitudes about the insanity defense.

**NGRI:** Participants were asked to rate the likelihood of rendering an NGRI verdict on a Likert scale of 1 (*extremely unlikely*) to 7 (*extremely likely*). Ratings for the current sample ranged from 1 to 7 (*M* = 4.10, *SD* = 1.99).

**Demographic Survey:** Participants were given a demographic survey to gather descriptive information about our sample. This demographic information included ethnicity, age, gender, political beliefs, education level, relationship status, and employment status.

### 10.3. Procedure

The procedure was identical to the pilot study aside from the additional measures described above (i.e., WCS, IDA-R, and NGRI endorsement). After completing the survey, participants received a debriefing explaining the purpose of the study.

## 11. Results

### 11.1. Data Screening and Attention Checks

Prior to conducting the primary analyses, we examined the data for failed attention checks. There were two attention checks in the Insanity Defense Attitudes—Revised Scale (IDA-R), and there was one in the Witness Credibility Scale (WCS). There was a total of four participants who failed at least two attention checks and were subsequently removed from the final data set. The remaining sample included 425 participants. Additionally, participants were asked the following information about the expert witness: number of years of education they had (high knowledge % correct = 69.2; low knowledge % correct = 81.3), number of years in the field (high knowledge % correct = 61.6; low knowledge % correct = 67.8), number of published research publications (high knowledge % correct = 44.1; low knowledge % correct = 17.8), number of forensic assessments administered (high knowledge % correct = 45.0; low knowledge % correct = 72.9), type of degree they had (high knowledge % correct = 79.1; low knowledge % correct = 79.9), and if they were board-certified (high knowledge % correct = 97.2; low knowledge % correct = 34.6).

### 11.2. Hypotheses Testing

Hypothesis 1 predicted that participants who were exposed to the high knowledge expert (Ph.D.) witness would view the expert as more credible than participants exposed to the low knowledge expert (M.S.). An independent samples t-test was performed to compare participants perceived credibility of the expert witness, between the expert witness knowledge conditions. Results indicated that there was not a significant difference in perceived credibility between the high knowledge (*M* = 151.99, *SD* = 28.41) and low knowledge condition (*M* = 149.41, *SD* = 29.26); *t*(423) = 0.92, *p* = 0.18, *d* = 0.09. However, after conducting an independent samples t-test on the WCS knowledge subscale separately (possible range of scores 5 to 50), there was a significant difference in perceived knowledge between the high knowledge (*M* = 38.99, *SD* = 7.56) and low knowledge conditions (*M* = 37.64, *SD* = 7.80); *t*(423) = 1.82, *p* = 0.035, *d* = 0.18. Additional *t*-tests were conducted on the likeability, trustworthiness, and confidence subscales, revealing no significant differences between conditions on any of the subscales, *p* > 0.05 (see Table 1 for statistics).

Hypothesis 2 predicted that participants exposed to the high knowledge expert (Ph.D.) witness would be more likely than those exposed to the low knowledge expert (M.S.) to endorse NGRI. An independent samples t-test was conducted to compare participants’ endorsement of NGRI between the two expert witness knowledge conditions. Results indicated that there was a significant difference in the likelihood of endorsing NGRI between the high knowledge (*M* = 3.92, *SD* = 2.00) and low knowledge conditions (*M* = 4.28, *SD* = 1.98); *t*(423) = −1.87, *p* = 0.031, *d* = 0.18. However, it was opposite of the predicted direction. Participants who read the low knowledge expert witness’ testimony were more likely to endorse NGRI than participants who read the high knowledge expert witness’ testimony. Thus, Hypothesis 2 was not supported.

To test Hypothesis 3, the analysis (as seen in Table 2 below) tested the main effects of expert witness knowledge and insanity defense attitudes, as well as their interaction on NGRI endorsement. The main effect of expert witness knowledge was not statistically significant in the model, *B* = −0.28, *t*(421) = −1.62, *p* = 0.107, 95% CI [−0.62, 0.06]. However, the main effect of insanity defense attitudes was statistically significant, *B* = −0.07, *t*(421) = –7.20, *p* < 0.001, 95% CI [−0.08, −0.05], suggesting that more negative attitudes toward the insanity defense were associated with lower levels of NGRI endorsement (*β* = −0.441), supporting Hypothesis 3. The interaction between expert witness knowledge and insanity defense attitudes was not significant, *B* = −0.01, *t*(421) = −0.42, *p* = 0.677, 95% CI [−0.03, 0.02].

To test Hypothesis 4, which predicted that participants in the high knowledge condition (*M* = 3.92, *SD* = 2.00, range = 3.65–4.19) would be more likely to endorse NGRI compared to those in the low knowledge condition (*M* = 4.28, *SD* = 1.98, range = 4.01–4.55), but only if those participants had lower IDA-R scores (i.e., positive or neutral attitudes toward NGRI), a moderation analysis was conducted. Specifically, Hayes’ PROCESS macro for SPSS (version 4.3.1; Hayes, 2023) was used to examine whether participants’ insanity defense attitudes (IDA-R) (high knowledge condition: *M* = 54.61, *SD* = 13.49, range = 52.78–56.44; low knowledge condition: *M* = 53.39, *SD* = 13.31, range = 51.60–55.19) moderate the effect of knowledge of the expert witness (high/low) on NGRI endorsement (PROCESS Model 1—See Table 2).^1^

## 12. Discussion

The aim of the current study was to examine the impact of expert witness knowledge on public perception of expert witness credibility and NGRI endorsement in the context of the insanity plea in the United States. We predicted that the participants who were exposed to the high knowledge expert witness would perceive the expert as more credible (H1) and would report increased NGRI endorsement (H2) compared to the participants exposed to the low knowledge expert witness. Additionally, we predicted that participants who have more positive attitudes towards the insanity defense (IDA-R) would be more likely to endorse NGRI than those who have negative attitudes (H3). Lastly, we predicted that the relationship between expert witness knowledge and NGRI endorsement would be moderated by the IDA-R. Specifically, we predicted that participants in the high knowledge condition and with more supportive attitudes towards the insanity plea (i.e., lower IDA-R scores) would be more likely to endorse NGRI compared to the participants in the low knowledge condition (H4). Our findings revealed support for H3, but failed to find support for H1, H2, and H4.

Past research has shown that the manipulation of expert witness credentials and testimony has resulted in increased perceived credibility of the expert witness as measured by the WCS (Ferreria & Wingrove, 2024; Greenberg & Wursten, 1988; Hurwitz et al., 1992; Koehler et al., 2016; LaBat et al., 2023; Neal et al., 2012b; Parrott et al., 2015a, 2015b; Younan & Martire, 2021). In the current study, there was not an impact of the knowledge manipulation (high vs. low) on overall credibility or any of the subscales except for knowledge (i.e., trustworthiness, likeability, and confidence). While the pilot study showed a significant difference in perceived knowledge, this difference did not impact expert witness credibility in the main study. Even with the pilot study findings, it is possible that the difference was not large enough to impact credibility. It is also possible that the trial context impacted credibility ratings. Given that this is the first study to examine these variables within an NGRI case, it is difficult to map our findings onto previous work. For example, Neal et al. (2012b) manipulated knowledge (high/low) and likeability (high/low) to examine the effect they had on expert witness credibility and found a significant positive relationship between expert witness knowledge and credibility; however, this was in the context of a capital murder case, which is substantially different from NGRI. Rather than focusing on the severity of the crime or sentencing outcomes, the focus of NGRI is instead on the defendant’s mental state and criminal responsibility at the time of the offense. These unique features of an NGRI verdict may influence how jurors process expert testimony by shifting the attention away from punishment towards psychological evaluation. Furthermore, the expert witness’ testimony in the current study was approximately 45 s, which may not have allowed adequate time for participants to fully evaluate its content (Parrott et al., 2015b; Titcomb, 2010). Given the lack of extensive testimony, participants may have defaulted to their own attitudes towards NGRI, a finding that would align with the previous literature (Titcomb, 2010). Additionally, the WCS subscales of likeability and trustworthiness are better portrayed by either video or audio testimony recordings, to capture the expert witness’ facial expression and body language. Past research has made use of this type of testimony, with expert witnesses being perceived as more relaxed, extroverted, and positive, receiving higher credibility ratings (Miller & Burgoon, 1982). Conversely, aspects of the expert witness, such as less eye contact and excessive gesturing, were associated with a lower credibility score. Unlike previous studies, such as Neal et al. (2012b), who made use of video testimony, this was outside the capabilities and scope of the current study. This could also explain why the WCS subscales of trustworthiness, likeability, and confidence were not impacted.

Unlike past research (Ferreria & Wingrove, 2024; Koehler et al., 2016; Parrott et al., 2015b), our study did find a relationship between expert witness knowledge and sentencing outcome. However, it was not in the expected direction. Participants exposed to the low knowledge expert witness were more likely to endorse NGRI compared to those exposed to the high knowledge expert. In other words, participants who read the testimony of the expert witness with significantly less experience and education (e.g., M.S.) endorsed NGRI more often than the participants exposed to the expert witness with a PhD and years of experience. One possible explanation for this finding is the hired gun effect. The hired gun effect is when an expert witness is perceived to be hired in a court case to favor the request of the retaining attorney rather than the evidence in the case in exchange for compensation (Ziemke & Brodsky, 2015). If mock jurors perceive the expert witness as being a hired gun, whether the motives are money, personal connections within the courtroom, or sympathy, then they will interpret that witness to be biased (Ziemke & Brodsky, 2015). This interpretation is applicable regardless of whether there are opposing experts (Ziemke & Brodsky, 2015). Another study found that mock jurors used expert witness’ pay and the frequency of testimonies (i.e., court appearances) as persuasion cues, but only when the testimony was complex (Cooper & Neuhaus, 2000). Furthermore, if mock jurors suspect that the expert witness is a hired gun, that will also reduce their perceived credibility, especially their trustworthiness and likeability (Cooper & Neuhaus, 2000), and subsequently increase guilty verdicts (Ziemke & Brodsky, 2015). In the current study, if the participants in the high knowledge condition identified the expert as a hired gun, even subconsciously, it would make sense that they would be more punitive in their verdict decisions by not endorsing NGRI. This bias could have been formed because of the expert’s extensive courtroom experience in the high knowledge condition (e.g., around 4000 forensic assessments with defendants and many court cases), mapping onto the results in Cooper and Neuhaus (2000). Additionally, the expert witness’ greater expertise may have backfired. For instance, if mock jurors expect the more experienced expert witness to present a strong argument but perceive that argument as weak, this may violate their expectations and lead them to reject the testimony, making them less likely to endorse NGRI. In other words, highly experienced experts who deliver weak arguments can reduce their ability to persuade by increasing scrutiny and skepticism, illustrating a backfire effect (Tormala et al., 2006).

These post hoc explanations also fit within the ELM framework. More specifically, if participants in the high knowledge condition perceived the expert as a hired gun (i.e., negative peripheral cue), then this heuristic processing may have overridden the testimony provided by the expert, resulting in lower NGRI endorsement. In contrast, participants in the low knowledge condition may have been less focused on source characteristics (e.g., how many times the expert had testified), and more on the testimony itself, encouraging central route processing. Similarly, if participants expected a strong argument from an expert with high credentials but perceived that expert’s testimony as weak, this expectancy violation may have undermined the persuasive power of that expert’s testimony. It is also possible that participant misconceptions about NGRI may have served as an extraneous variable. Perhaps the ambiguity and lack of knowledge of what an NGRI verdict entails for the defendant (e.g., what happens after the verdict; Finkel, 1995) may have driven verdict decisions.

To contextualize the findings of Hypotheses 1 and 2, it is important to address the practical significance of these results. Although the statistical differences were modest, in the real world, these subtle changes could have a notable impact on public attitudes. For instance, in the current study, participants were briefly exposed to case information and expert credentials, similar to the exposure individuals experience through the news media. Even with this minimal exposure, we still observed movement on NGRI endorsement, suggesting that small changes in perceived knowledge can shift perceptions of NGRI. Given that insanity defense cases are often high-profile due to their rarity and egregiousness, exposure to case information is likely to be repetitive and prolonged in the news cycle. Thus, while the effects we observed are small, they could theoretically be much larger in real life (i.e., cumulative effect).

Regarding attitudes toward the insanity defense, results demonstrated that individuals with more positive attitudes towards NGRI (i.e., higher scores on the IDA-R) were more likely to endorse it. This finding aligns with previous scholarship. For instance, research has shown that, in general, those with strong opinions towards a particular sentencing outcome (Skeem et al., 2004) are less likely to be swayed by expert witness testimony compared to those who may be on the fence or unsure of their position/stance. Although the IDA-R measures attitude valence (positive vs. negative) towards the insanity defense rather than attitude strength, recent research suggests that attitude attributes such as ambivalence and certainty are strong predictors of attitude strength in that increased certainty predicts increased strength of the attitude (Luttrell & Sawicki, 2020). Thus, more extreme scores on the IDA-R (i.e., stronger agreement or disagreement) could serve as a proxy for attitude strength, suggesting participants may have relied more on central route processing (e.g., pre-existing attitudes), and consequently, experienced greater resistance to persuasion, consistent with the elaboration likelihood model (ELM). However, we acknowledge that determining individual-level processing is complex; therefore, scholars should further explore ELM in the context of the insanity defense by incorporating a more direct measure of attitude strength, such as assessing resistance to counterarguments and stability over time (Luttrell & Sawicki, 2020). This could be performed in a lab setting by measuring individuals’ attitudes, exposing them to beliefs or arguments that counter their attitudes, and evaluating their beliefs post-exposure. The degree to which their attitudes change would indicate their attitude strength. Additionally, the demographics of the current sample seemed to match with what previous research has found for individuals who are more likely to endorse an NGRI verdict, in that most participants were college educated and were mostly of a Liberal political affiliation (Butler, 2006). In the referenced study, 77% of their participants had at least some college education. Additionally, 49% of liberal and 41% of slightly liberal participants chose an NGRI verdict, compared to 24% of conservative participants who also chose the same verdict (Butler, 2006).

### Implications

Although we only found partial support for our hypotheses, these findings have implications for the courtroom. For instance, when jury selection is underway for a capital sentencing trial, jurors undergo a qualification process, during which their stance on capital punishment is assessed (Colby, 2020). Jurors must be “death qualified,” that is, they should not strongly oppose capital punishment, while also agreeing that it should not be used in all capital murder cases. However, jurors serving on NGRI cases do not undergo a qualification process. Given the significant link between attitudes toward the insanity defense and NGRI endorsement, jurors may need to undergo “NGRI qualification” to prevent bias similar to “death qualification” in capital cases. However, death qualification has raised some concerns and appears to be an imperfect solution. For instance, Haney et al. (2022) found that death-qualified jurors across three U.S. states were more punitive in verdict decisions, less informed (i.e., the belief that the death penalty deters murder and is cheaper than a life sentence), less concerned with fairness of mistakes (i.e., wrongful convictions, methods being painful/cruel), and less likely to consider mitigating factors during sentencing. Further, death qualification systematically excludes those who are most skeptical of the death penalty, which, as a result, means that capital juries may consist of individuals who are less informed and reflective in their decisions. Despite these findings, whether death qualification violates the rights of the Sixth Amendment is debated among legal scholars. For instance, Colby (2020) discusses death qualification from an originalist standpoint and highlights that even though it does not seem to violate the Constitution directly, death qualification removes a large portion of society from jury service, particularly those with moral or religious objections to the death penalty. This raises concern about a lack of diverse jury representation, which inadvertently produces ideologically skewed juries. Therefore, while an NGRI qualification process may help ensure that jurors are willing to consider an NGRI verdict, it may paradoxically introduce more bias into the jury. Future research should explore qualified samples to determine whether a qualification process would reduce or exacerbate bias in NGRI trials.

## 13. Limitations and Future Directions

While the current study is the first to explore expert credibility in the context of NGRI, it is not without limitations. One limitation was the lack of audio or video testimony. Although manipulations of knowledge can occur with written testimony, it is difficult to portray aspects of credibility that are more dependent on visual cues, such as likeability and confidence (e.g., facial expressions and body language). This likely contributed to the absence of differences on the overall WCS. Another explanation could be that both experts were highly qualified. We purposefully chose graduate-level experts to maintain ecological validity, but this may have reduced the salience of our manipulation. Nevertheless, participants still perceived a difference in knowledge as reflected on the knowledge subscale, suggesting the manipulation was noticeable, albeit subtle. Future research should test other ways of highlighting differences in expertise, such as institutional prestige (e.g., Cooper & Neuhaus, 2000).

A second limitation was the lack of expansive trial materials. Although the goal of the study was to explore public perceptions rather than jury verdicts, incorporating full jury instructions, cross-examination, opposing experts, and deliberation could alter findings. Notably, participants in the low knowledge condition were more likely to endorse NGRI, counter to prior research (Ferreria & Wingrove, 2024; Koehler et al., 2016; Parrott et al., 2015b). Future studies should examine whether trial elements, such as dispositional instructions about the consequences of an NGRI verdict (e.g., Wheatman & Shaver, 2001; Kanani, 2024; Raker, 2023), influence these outcomes, particularly in more ambiguous cases.

A third limitation was the use of an unambiguous case summary modeled after Hudachek and Quigley-McBride (2022). Because the defendant’s mental health was clearly at issue, participants likely relied on their preexisting attitudes rather than expert credentials. This helps explain why differences did not emerge on the overall WCS. According to the ELM framework, individuals are more likely to rely on peripheral cues if information is ambiguous or complex (Petty & Cacioppo, 1986). Thus, future research should employ more ambiguous case summaries to test whether attitudes moderate the effect of expertise. At the same time, using a brief and straightforward case mirrors the limited information the public often receives through media coverage of high-profile trials. Even such minimal exposure can shape perceptions of fairness and justice, which are central to procedural justice and can ultimately influence views of legitimacy and obedience to the law (Tyler, 2003).

Finally, reliance on a single case vignette limits the generalizability but increases internal validity through greater experimental control (Aguinis & Bradley, 2014). Single-vignette designs are common in legal psychology research (e.g., Ferreria & Wingrove, 2024; Flick et al., 2022; Parrott et al., 2015b) and provide a solid foundation for future investigations. Nonetheless, incorporating stimulus sampling in future studies (see Wells & Windschitl, 1999) will help establish boundary conditions and enhance construct validity.

## 14. Conclusions

The current study examined the impact of expert witness knowledge on perceived credibility and NGRI endorsement. Although past research has demonstrated that expert witness credentials influence juror perceptions of credibility, the current study did not replicate these findings. Instead, results indicated that individual attitudes toward the insanity defense were the strongest predictor of NGRI endorsement, outweighing the influence of expert witness-related factors such as testimony, credentials, or experience. Given the ambiguous nature of the insanity defense and the complexity of legal standards for criminal responsibility, individual attitudes may have a more central role in shaping decision-making than the perceived credibility of expert witnesses. While this study offers a novel contribution to the existing literature through the exploration of expert witness credibility in the context of NGRI, the findings should be examined further in subsequent studies. Overall, the implications of this initial investigation suggest that juror preexisting attitudes may exert a greater influence on verdict outcomes, at least in the current case. These results highlight the need to account for juror attitudes when evaluating the impact of expert witness testimony in criminal trials involving the insanity defense.

## Figures and Tables

**Table 1:** Hypothesis 1—*t*-tests for Witness Credibility Scale subscales.

**Subscale**	**High Knowledge Condition *M* (*SD*)**	**Low Knowledge Condition *M* (*SD*)**	***t***	***p***	**Cohen’s *d***
**Likeability**	33.70 (7.19)	34.23 (7.57)	−0.75	0.227	0.073
**Knowledge**	39.00 (7.56)	37.64 (7.80)	1.82	0.035	0.18
**Trustworthiness**	38.26 (9.14)	37.52 (8.65)	0.843	0.200	0.082
**Confidence**	39.46 (7.40)	38.33 (7.26)	1.56	0.057	0.154

*Note:* All *p*-values are one-tailed.

**Table 2:** Hypothesis 4—statistics for moderation model.

Predictor	*b*	*SE*	*t*	*p*	95% CI
**Constant**	4.24	0.12	35.02	<0.001	[4.00, 4.48]
**Condition (Expert Witness Knowledge)**	−0.28	0.17	−1.62	0.107	[−0.62, 0.06]
**IDA_SUM**	−0.07	0.01	−7.20	<0.001	[−0.08, −0.05]
COND*IDA_SUM	−0.01	0.01	−0.42	0.677	[−0.03, 0.02]

Note: *b* = unstandardized regression coefficient; *SE* = standard error; CI = confidence interval.

## Data Availability

The raw data supporting the conclusions of this article will be made available by the authors on request.

## Note

1 Two separate models were also analyzed containing each IDA-R subscale separately. These models did not significantly differ from the model with the total IDA-R.

## Appendix A

### Case Summary

Ralph Brown is a 33-year-old male diagnosed with schizophrenia and has an extensive history of hospitalizations due to his mental illness. Mr. Brown has been known to talk to himself in public and believes that voices from the radio or TV direct him to do things on a daily basis. He and his ex-girlfriend of two years, Sandra, broke up a few months ago, and she began dating another man soon after. On the night of 23 October 2018, Mr. Brown allegedly broke into the apartment that Sandra and her boyfriend shared while they were asleep and shot the boyfriend in the head, killing him instantly.

The prosecution argues that Mr. Brown was unable to accept that Sandra had moved on and was happy. It was discovered that the gun used to murder Sandra’s boyfriend is registered under Mr. Brown, which was purchased soon after the breakup. Due to his strong feelings for Sandra and the time frame in which Mr. Brown purchased the gun give the prosecution reason to believe that Mr. Brown had premeditated intent to harm. Mr. Brown was in a clear enough state of mind to know that he was taking a life and is ultimately responsible for the murder.

The defense claims Mr. Brown loved Sandra deeply and that prior to going to the apartment, voices on the TV told him that Sandra’s current boyfriend was cheating on her and to “get rid of him.” Mr. Brown testified at trial that the voices were trying to protect Sandra from getting hurt and that he was the only one who could protect her. Upon further investigation, it was discovered that Mr. Brown had recently stopped taking his antipsychotic medication, which led to his psychotic break and, ultimately, the murder.

At trial, a forensic psychologist testified for the defense. The following page contains their testimony.

## Appendix B

### Appendix B.1. Expert Witness Knowledge Manipulation

#### Appendix B.1.1. High Knowledge Condition

Expert Witness Credentials: The forensic psychologist went to school for 10 years, has her doctorate degree (Ph.D.), is board-certified by the American Board of Forensic Psychology, and has been in the profession for over 20 years. She has administered about 4000 forensic assessments and published 50 research publications. She has also testified in many court cases and conducted many interviews with defendants prior to testifying.

#### Appendix B.1.2. Low Knowledge Condition

Expert Witness Credentials: The forensic psychologist went to school for 6 years, has her master’s degree (M.S.) and has been working in her profession for 2 years. She has conducted around 100 forensic assessments and has 4 research publications. She has testified in a handful of court cases and conducted a few interviews with defendants prior to testifying.

## Appendix C

### Testimony

“Mr. Brown’s symptoms of schizophrenia appear to impact his ability to distinguish between what is reality and what is imagined, which is evidenced by his delusions. Specifically, referential delusions make people believe that everyday events, objects, or actions have a special and personal significance to them. In Mr. Brown’s case, he believes that the voices from the TV or radio are actually speaking to him and directing him to engage in certain behaviors. A characteristic of delusions of this nature is that an individual with schizophrenia often has difficulty and is frequently unable to distinguish between their own thoughts and the thoughts being implanted in them without their consent. In an interview conducted with Mr. Brown, he indicated that the voices had never directed him to do anything violent until the night of the crime and that before that night, he had never been extremely alarmed by these voices. During our conversation, Mr. Brown exclaimed that he loved Sandra and was only trying to protect her from finding out that she was being cheated on. Mr. Brown stated that Sandra is the only person in his life who made him feel safe, and he wanted to protect her from any harm. Due to this way of thinking, Mr. Brown does not understand that killing Sandra’s boyfriend is wrong and did not commit this act during a time when thinking clearly or rationally and should thus necessitate psychiatric help.”

## Appendix D

### Appendix D.1. Main Study Measures

1. Witness Credibility Scale: Please rate the expert witness for the following items on the scale provided (i.e., not at all friendly (1) and extremely friendly (10))
  If you are unsure, please make your best guess.
  - Friendly
  - Respectful
  - Kind
  - Manner
  - Pleasant
  - Trustworthy
  - Truthful
  - Dependable
  - Honest
  - Reliable
  - Confident
  - Well-spoken
  - Self-assured
  - Informed
  - Logical
  - Educated
  - Wise
  - Scientific
  - Poised
  - Relaxed
2. Insanity Defense Attitude-Revised (IDA-R): Please answer these questions to the best of your ability on the following scale 1 (disagree completely) to 5 (agree completely).
3. We should punish people who commit criminal acts, regardless of their degree of mental disturbance.
4. I believe that people should be held responsible for their actions no matter what their mental condition.
5. Mentally ill defendants who plead insanity have failed to exert enough willpower to behave properly like the rest of us. So, they should be punished for their crimes like everyone else.
6. I believe that all human beings know what they are doing and have the power to control themselves (moderate load on both factors).
7. A defendant’s degree of insanity is irrelevant: if he commits the crime, then he should do the time.
8. With slick attorneys and a sad story, any criminal can use the insanity defense to finagle his way to freedom.
9. As a last resort, defense attorneys will encourage their clients to act strangely and lie through their teeth to appear “insane.”
10. The insanity defense threatens public safety by telling criminals that they can get away with a crime if they come up with a good story about why they did it.
11. For the right price, psychiatrists will probably manufacture a “mental illness” for any criminal to convince the jury that he is insane.
12. The insanity defense returns disturbed, dangerous people to the streets.
13. The insanity plea is a loophole in the law that allows too many guilty people to escape punishment
14. Many of the crazy criminals that psychiatrists see fit to return to the streets go on to kill again.
15. It is wrong to punish people who commit crime for crazy reasons while gripped by uncontrollable hallucinations or delusions (reverse scored).
16. Some people with severe mental illness are out of touch with reality and do not understand that their acts are wrong. These people cannot be blamed and do not deserve to be punished.
17. I believe that we should punish a person for a criminal act only if he understood the act as evil and then freely chose to do it (reverse scored).
18. I believe that mental illness can impair people’s ability to make logical choices and control themselves (reverse scored).
19. It is wrong to punish someone for an act they commit because of any uncontrollable illness, whether it be epilepsy or mental illness (reverse scored).
20. Most defendants who use the insanity defense are truly mentally ill, not fakers (reverse scored).
21. Perfectly sane killers can get away with their crimes by hiring high-priced lawyers and experts who misuse the insanity defense.
22. NGRI Endorsement: The following is a definition of not guilty by reason of insanity plea in South Carolina: “Every man is to be presumed to be sane, and …that to establish a defense on the ground of insanity, it must be clearly proved that, at the time of the committing of the act, the party accused was laboring under such a defect of reason, from disease of mind, and not to know the nature and quality of the act he was doing; or if he did know it, that he did not know he was doing what was wrong” Please rate how likely you are to give a verdict of not guilty by reason of insanity (1—extremely unlikely; 7—extremely likely)
  - Not guilty by reason of insanity (NGRI): 7-point scale: 1 = extremely unlikely, 7 = extremely likely
23. What was your perception of the knowledge of the expert witness in the case you read?
24. 7-point scale: 1 = not at all knowledgeable, 7 = extremely knowledgeable

### Appendix D.2. Demographic Questions

25. What is your age?
  - Options: qualitative response
26. What racial or ethnic group best describes you?
  - Options: Asian, Black, Hispanic/Latino, Middle Eastern, Native American, White, Two or more races, other
27. What is your gender?
  - Options: male, female, non-binary/non-conforming, transgender, other, prefer not to answer
28. In general, how would you describe your political beliefs?
  - Options: very liberal, liberal, moderate, conservative, very conservative
29. What is the highest level of education you have completed?
  - Options: high school graduate; some college, but no degree (yet); 2-year college degree, 4-year college degree, postgraduate degree
30. What is your relationship status?
  - Options: married, living with spouse; separated, divorced, widowed, single, never married; domestic partnership
31. What is your current employment status?
  - Options: full time job, part time job, temporarily laid off, unemployed, retired, permanently disabled, taking care of home or family, student, other
32. Are you a U.S. citizen?
  - Options: yes or no

Attention Checks:

33. Select the number 8 for this question.
  - Options: 1, 2, 3, 4, 5, 6, 7, 8, 9, 10
34. Select the number 2 for this question.
  - Options: 1, 2, 3, 4, 5
35. Select the number 4 for this question.
  - Options: 1, 2, 3, 4, 5
